# The comprehensive complication index (CCI): proposal of a new reporting standard for complications in major urological surgery

**DOI:** 10.1007/s00345-020-03356-z

**Published:** 2020-08-19

**Authors:** K. F. Kowalewski, D. Müller, J. Mühlbauer, J. D. Hendrie, T. S. Worst, F. Wessels, M. T. Walach, J. von Hardenberg, P. Nuhn, P. Honeck, M. S. Michel, M. C. Kriegmair

**Affiliations:** 1 Department of Urology and Urological Surgery, University Medical Center Mannheim, University of Heidelberg, Theodor-Kutzer-Ufer 1-3, 68167 Mannheim, Germany; 2 Department of Internal Medicine, Prisma Health, 701 Grove Road, Greenville, SC 29605 USA

**Keywords:** Complications, Comprehensive complication index, Clavien dindo classification, Urology, Surgery

## Abstract

**Purpose:**

The comprehensive complication index (CCI) is a new tool for reporting the cumulative burden of postoperative complications on a continuous scale. This study validates the CCI for urological surgery and its benefits over the Clavien-Dindo-Classification (Clavien).

**Material and methods:**

Data from a prospectively maintained data base of all consecutive patients at a university care-center was analyzed. Complications after radical cystectomy (RC), radical prostatectomy (RP), and partial nephrectomy (PN) were classified using the CCI and Clavien system. Differences in complications between the CCI and the Clavien were assessed and correlation analyses performed. Sample size calculations for hypothetical clinical trials were compared between CCI and Clavien to evaluate whether the CCI would reduce the number of required patients in a clinical trial.

**Results:**

682 patients (172 RC, 297 RP, 213 PN) were analyzed. Overall, 9.4–46.6% of patients had > 1 complication cumulatively assessed with the CCI resulting in an upgrading in the Clavien classification for 2.4–32.4% of patients. Therefore, scores between the systems differed for RC: CCI (mean ± standard deviation) 26.3 ± 20.8 vs. Clavien 20.4 ± 16.7, *p* < 0.001; PN: CCI 8.4 ± 14.7 vs. Clavien 7.0 ± 11.8, *p* < 0.001 and RP: CCI 5.8 ± 11.7 vs. Clavien 5.3 ± 10.6, *p* = 0.102. The CCI was more accurate in predicting LOS after RC than Clavien (*p* < 0.001). Sample size calculations based in the CCI (for future hypothetical trials) resulted in a reduction of required patients for all procedures (− 25% RC, − 74% PN, − 80% RP).

**Conclusion:**

The CCI is more accurate to assess surgical complications and reduces required sample sizes that will facilitate the conduction of clinical trials.

**Electronic supplementary material:**

The online version of this article (10.1007/s00345-020-03356-z) contains supplementary material, which is available to authorized users.

## Introduction

Surgery remains the mainstay of treatment for localized urological cancers. Through the ongoing development of new surgical innovations (e.g., robotic techniques), highly complex procedures can generally be carried out in a safe and standardized manner. However, postoperative complications are inevitable, especially when elderly and multimorbid patients are deemed candidates for surgery [1, 2]. The objective assessment of complications is essential in order to minimize systematic errors and optimize patient care. The Clavien-Dindo-Classification (Clavien) is the present standard to assess perioperative morbidity and mortality [3]. It classifies complications into five grades based on increasing severity, with grade 5 indicating patient death. A strength of the Clavien system is, that its classification is based on the invasiveness of the required treatment thereby taking into account that a certain complication might present with a different severity. Although originally designed for general surgery, the Clavien system was additionally validated by the European Association of Urology (EAU) for evaluating postoperative complications of urological surgeries [4]. Despite its validation, the Clavien system has considerable drawbacks that limit its readability and interpretation. For instance, it is reported as ordinal data, which limits its ability to comprehensively compare two treatment options (e.g., open vs. laparoscopic surgery) as each grade must be considered separately in the absence of a summative interpretation. Furthermore, the absence of a weighting system restricts cross-grade comparison (e.g., inability to compare three grade I complications vs. one grade III complication). Moreover, several studies have shown that often only the highest grade complication is captured [5–7], which leads to data loss in patients who have both low- and high-grade complications. In order to account for the aforementioned limitations, the *comprehensive complication index* (CCI)—not to be confused with the Charlson’s Comorbidity index—was developed and is now the reported standard in general surgery [8]. The CCI is based on the Clavien system but accounts for all accumulated complications and provides a continuous overall score between 0–100 (where 100 would indicate patient death).

Despite its acceptance in general surgery, the CCI is not yet validated for urological procedures. Despite Vetterlein et al. and Furrer et al. applied the CCI to report on their respective patient cohorts undergoing radical cystectomy (RC) [9, 10], the CCI has not been used or validated for other major uro-oncological procedures such as radical prostatectomy (RP) or partial nephrectomy (PN). Therefore, the aim of the presented study was to introduce and validate the CCI for the three most common uro-oncological procedures (RC, RP and PN) using a patient cohort from a tertiary university referral center, and to analyze the CCI data distribution for these procedures.

## Material and methods

### Patient population

Patient information was retrieved from prospectively-maintained databases for RC, RP and PN (institutional review board approval 2015-549 N-MA & 2014-811R-MA). Therefore, it should be acknowledged, that we did not start a prospective data collection at the beginning of the study, but reviewed our database. For the RC and PN cohorts, all patients between January 1st, 2017 and December 31st, 2018 were considered. For PN, patients who had bilateral tumors or additional procedures at the same time were excluded from analysis. For RP, patients between January 1st, 2018 and December 31st, 2018 were considered. The time frames differ because prospective data acquisition was established at different points of time at our department. Only events that occurred during the hospital stay or within 30 days of the initial procedure were considered.

### Assessment of complications

Data was extracted and entered into a dedicated spreadsheet which listed all complications for each patient (noting the highest-grade complication based on the Clavien classification). For each of the three data bases (RC, PN, RP) an urologist from our department is responsible for the quality and integrity of the data. Before the data bases were established, the complications were defined based on the EAU proposal. This also guaranteed consistency during the assessment. With this data a CCI score was generated for each patient using the freely available online tool www.assesssurgery.com. As mentioned before, the CCI provides a continuous scale between 0 and 100 (100 indicating death of a patient). To this end, each of the Clavien grades is assigned a specific CCI value and weight of complication (wC) as follows:

$$ {\mathbf{Clavien}} - {\mathbf{I}}: \to {\text{CCI score 8}}.{7} \to {\text{wC1}} = {3}00 $$

$$ {\mathbf{Clavien}} - {\mathbf{II}}: \to {\text{CCI score 2}}0.{9} \to {\text{wC1}} = {175}0 $$

$$ {\mathbf{Clavien}} - {\mathbf{IIIa}}: \to {\text{CCI score 26}}.{2} \to {\text{wC1}} = {275}0 $$

$$ {\mathbf{Clavien}} - {\mathbf{IIIb}}: \to {\text{CCI score 33}}.{7} \to {\text{wC1}} = {455}0 $$

$$ {\mathbf{Clavien}} - {\mathbf{IVa}}: \to {\text{CCI score 42}}.{4} \to {\text{wC1}} = {72}00 $$

$$ {\mathbf{Clavien}} - {\mathbf{IVb}}: \to {\text{CCI score 46}}.{2} \to {\text{wC1}} = {855}0 $$

$$ {\mathbf{Clavien}} - {\mathbf{V}}: \to {\text{always results in a CCI score of 1}}00 $$

The overall CCI score is then calculated with the formula:

$$ {\text{CCI}} = \frac{{\sqrt {{\text{wC}}1 + {\text{wC}}2 \ldots + {\text{wC}}x} }}{2} $$

Finally, in order to adhere with the EAU guidelines for reporting and grading complications, detailed information on mortality was additionally reported [11].

### Surgical techniques

Characteristics of the reported procedures (number per year, surgical approach, type of urinary diversion) are summarized in Table 1. At our department, all procedures are overseen by an experienced attending who is specialized in uro-oncologic surgery (> 100 procedures). Minimally invasive procedures are performed with robotic-assistance and conventional laparoscopy is not performed. RC is always performed with an open approach. Finally, RP and RC were performed with concomitant lymphadenectomy.

**Table 1:** Baseline characteristics of included patients

	Year	No. patients	Age (years)^a^	BMI (kg/m^2^)^a^	Gender (male)^b^	Surgical approach (open)^b^	Length of stay (days)^c^
Radical prostatectomy	2018	297	65.8 ± 7.2	27.2 ± 4.0	297 (100)	15 (5.1%)	7 (7–7)
Partial nephrectomy	2017–2018	213	63.1 ± 12.2	27.1 ± 5.3	146 (68.5)	167 (78.4%)	7 (6–8)
						Type of urinary diversion ^b^	
Open radical cystectomy	2017–2018	172	67.9 ± 11.0	26.8 ± 5.1	132 (76.7)	Ileal NB: 61 (35.5) IC: 86 (50.0) CU: 16 (9.3) Other: 9 (5.2)	16 (12–23)
Overall	2017–2018	682	65.5 ± 10.1	27.1 ± 4.7	576 (84.3)	n.a	10.7 ± 7.8

*IC* Ileal conduit, *NB* Neobladder, *n* number, *SD* standard deviation, *CU* Cutaneous ureterostomy

^a^Values reported as mean ± SD if not indicated otherwise

^b^Values reported as n (percentage) if not indicated otherwise

^c^Values reported median + interquartile range

### Statistical analysis

Patient characteristics such as age, gender, body mass index (BMI), surgical approach, and length of hospital stay (LOS) were extracted and reported with descriptive statistics (mean ± standard deviation (SD), relative and absolute frequencies). Scatter and bar plots were created to easily visualize the data. The Shapiro-Wilks test was performed and Quantile–Quantile-Plots (Q-Q-plots) were created to test for normality and thereby analyze whether parametric or non-parametric testing should be applied in future studies [12]. Parametric tests were applied to normally distributed data. Otherwise, non-parametric testing was performed using the Wilcoxon–Mann–Whitney test for group comparisons and Spearman’s rank correlation coefficient. Sample size calculation for a fictive future superiority trial was based on the assumption of a 30% reduction for complication incidence (yes vs. no) on the Clavien scale. As done elsewhere, sample size calculation using the CCI was performed using Noether’s formula [13] and made under the assumption that a ten point reduction in score for the RC cohort would reflect clinical relevance [14]. However, due to the small overall number of complications for RP and PN a delta of five points was assumed. The SD values were also estimated from the presented RC, RP, and PN cohorts, respectively. All statistical analyses were performed with *R* [15] using an alpha level of 0.05 and power of 0.80.

## Results

Overall there were 682 included patients, of which 172 underwent open RC (2017–2018), 213 underwent PN (2017–2018), and 297 underwent open or robotic-assisted RP (2018). Baseline characteristics can be found in Table 1.

### Complications and additional value of the CCI compared to the Clavien system

**Fig. 1:**
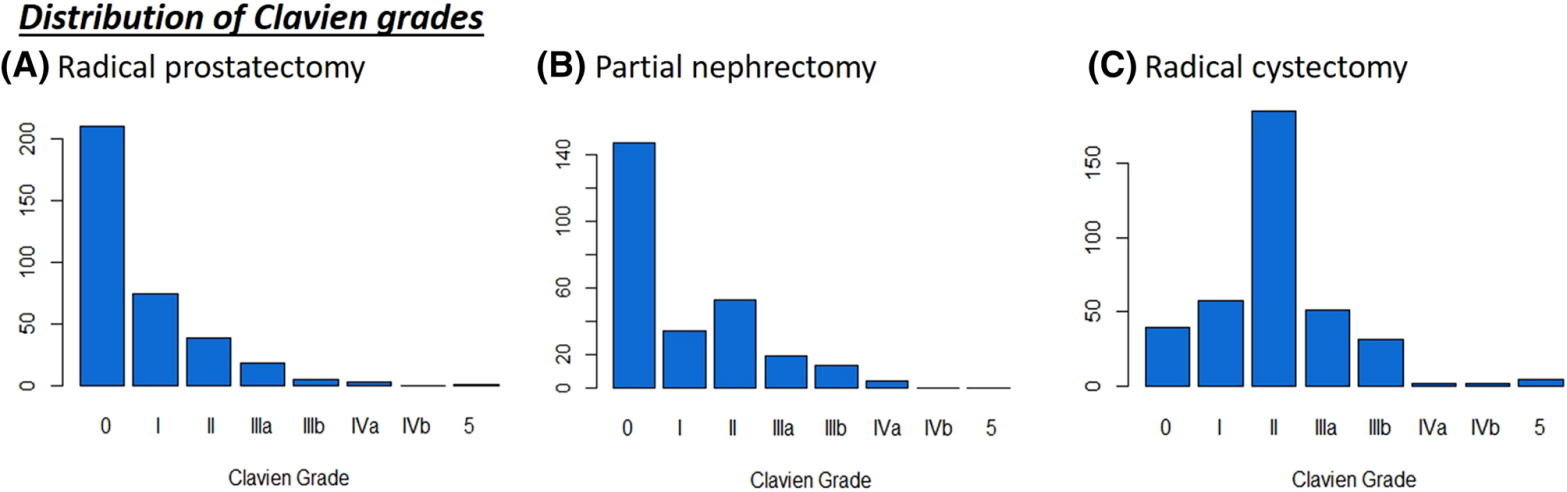
Distribution of complications stratified by Clavien grade among all procedures

The total number of complications stratified by the Clavien system can be found in Fig. 1. The overall complication rate was low for RP and PN while RC was associated with considerable morbidity. For RC, the Clavien grades were as followed: 52 grade I, 185 grade II, 51 grade IIIa, 31 grade IIIb, 1 grade IVa, 1 grade IVb and 4 grade V complications. For PN, the Clavien grades were: 34 grade I, 53 grade II, 19 grade IIIa, 13 grade IIIb and 4 grade IVa complications. Finally, for the RP cohort there were 74 grade I, 38 grade II, 18 grade IIIa, 5 grade IIIb, 3 grade IVa and 1 grade V Clavien complication. Furthermore, six out of the 172 RC patients developed wound complications.

**Fig. 2:**
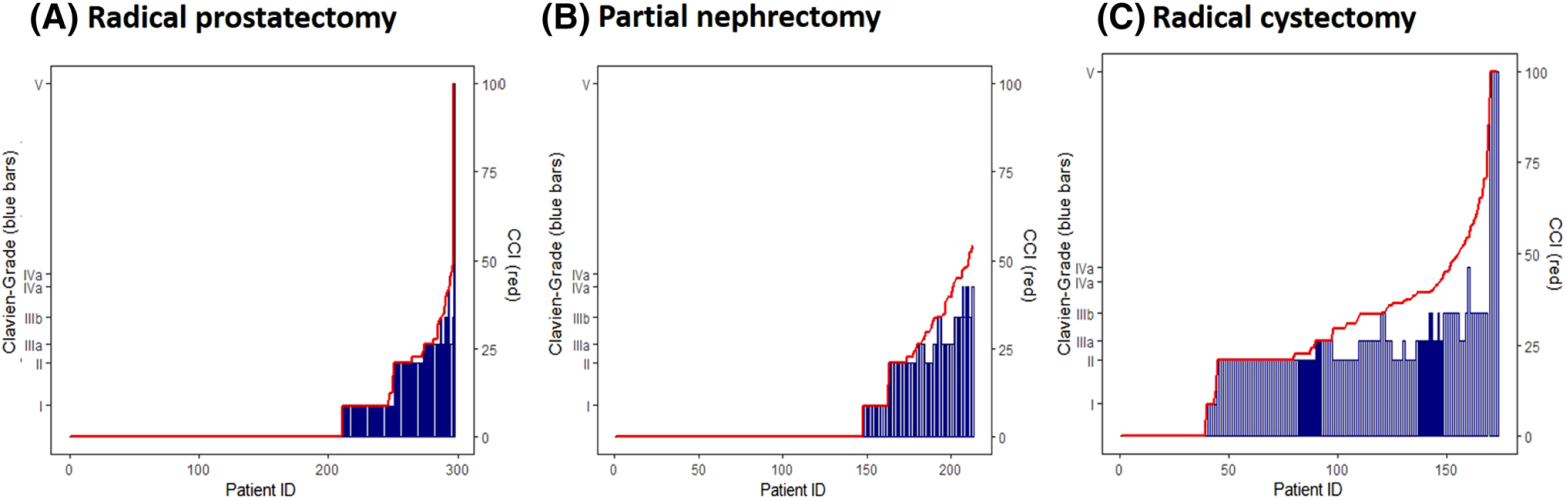
Comparison of the highest Clavien–Dindo Grade (blue bars) with the cumulative comprehensive complication index (red line) for each patient

Figure 2 depicts the complication burden with the adoption of the CCI compared to the traditional Clavien grading. Since the CCI summarizes and accounts for complications of various grades rather than just the most severe, 56 (32.4%) patients in the RC cohort ended up with a CCI score that correlated with a higher Clavien grade than their highest-grade complication alone. For the RP and PN cohorts, seven (2.4%) and 24 (11.3%) patients were also re-classified with a higher Clavien grade, respectively. The five most common complications by grade for each procedure can be found in Table 2. Overall the CCI accounted for relevant data that may have not been analyzed using the traditional Clavien system, as a total of 84 (48.6%) RC patients, 28 (9.4%) RP patients, and 34 (16.0%) PN patients experienced more than one complication. One death occurred in the RP cohort due to an unwitnessed intraoperative bowel perforation during a robotic-assisted RP, which led to peritonitis and small and large bowel ischemia. A total of 5 deaths occurred in the RC cohort: one from gastric ulcer bleeding, one from tumor progression with brain metastases, one from anastomotic leakage, one from pneumonia and one from sepsis.

**Table 2:** Most common postoperative complications within 30 days

	CDC grading	Management	Number of complications	Proportion in %
Radical cystectomy				
Anemia requiring transfusion	II	Blood transfusion	62	35.8
Urinary tract infection	II	Anti-infectious agents, Transurethral catheter insertion	17	9.8
	IIIa	Anti-infectious agents, Transurethral catheter, Cystoscopy/Image-guided transurethral catheterization	1	0.6
Acute kidney injury/hydronephrosis	II	Anti-infectious agents	2	1.2
	IIIa	Nephrostomy; Transurethral catheter; Anastomotic incision	16	9.2
Ileus	II	Gastric tube; Medical stimulation; Anti-infectious agents	8	4.6
	IIIb	Re-Laparotomy	4	2.3
Vomiting, Nausea	I	Anti-emetic treatment	1	0.6
	II	Gastric tube; Medical stimulation, Anti-emetic treatment	10	5.8
Radical prostatectomy				
Anastomotic leakage	I	Prolonged transurethral catheter	24	8.1
Lymphocele	IIIa	Drainage; Anti-infectious agents	17	5.7
Urinary tract infection	II	Anti-infectious agents, Transurethral catheter insertion	12	4.0
Anemia requiring transfusion	II	Blood transfusion	6	2.0
Urinary retention	I	Transurethral catheter insertion	6	2.0
Partial nephrectomy				
Anemia requiring transfusion	II	Blood transfusion	14	6.6
Pneumothorax	I	Conservative treatment	9	4.2
	IIIa	Thoracic drainage	4	1.9
AV-Fistula	IIIa	Coiling; Blood transfusion	9	4.2
Hematoma	I	Conservative treatment	5	2.3
	IIIb	Operative Revision	3	1.4
Pneumonia	II	Anti-infectious agents	7	3.3

### Data distribution of the CCI

Statistical testing via the Shapiro–Wilks test revealed a non-normal data distribution with significant results for RC, RP, and PN (*p* < 0.001 for each). In addition, Q-Q-plots (Supplementary) and visualization of the data distribution confirmed these findings (Fig. 1).

### Correlation with length of stay

**Fig. 3:**
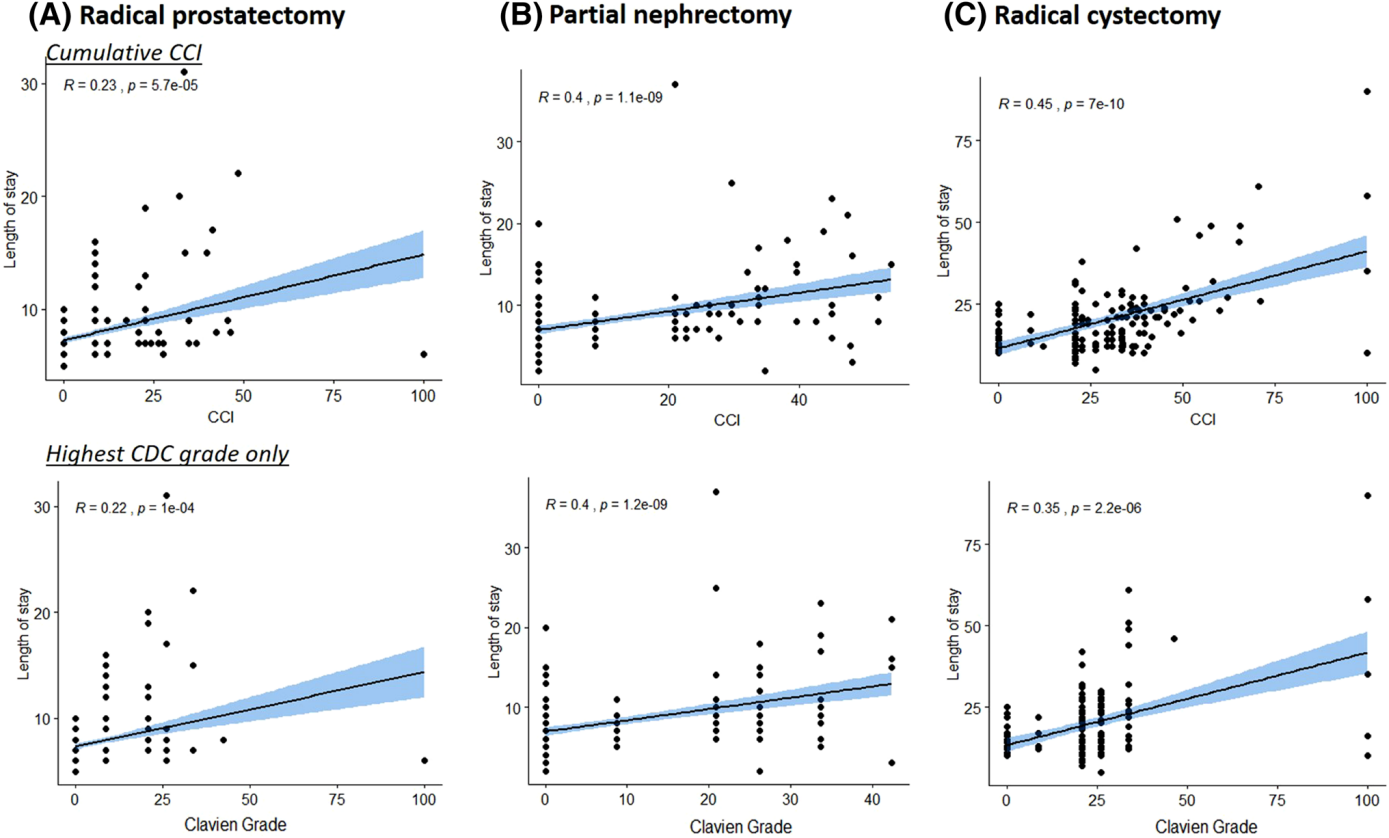
Correlation of length of stay with the cumulative CCI and non-cumulative Clavien

Comparing the correlation between cumulative CCI and LOS versus the Clavien system and LOS showed that the CCI enabled a more accurate prediction of LOS for the RC cohort (CCI: *r* = 0.45; *p* < 0.001 vs. Clavien: *r* = 0.35; *p* < 0.001; statistical difference between correlations *p* < 0.001) while correlation indices for RP (CCI: *r* = 0.23; *p* < 0.001 vs. Clavien: *r* = 0.22; p < 0.001; statistical difference between correlations *p* = 0.213) and PN (CCI: *r* = 0.4; *p* < 0.001 vs. Clavien: *r* = 0.4; *p* < 0.001; statistical difference between correlations *p* = 0.999) were not significantly different from each other (Fig. 3).

### Group comparisons and sample size calculation

Comparing the overall complication burden of the CCI with the Clavien syste, resulted in a significant difference for the RC (CCI mean ± standard deviation; [CI] 26.3 ± 20.8; [23.5–30.0] vs. Clavien: 20.4 ± 16.7; [18.2–23.6], *p* < 0.001) and PN cohorts (CCI: 8.4 ± 14.7; [6.4–10.4] vs. Clavien 7.0 ± 11.8; [5.4–8.6], *p* < 0.001). However, there were no differences for the RP cohort (CCI: 5.8 ± 11.7; [4.5–7.2] vs. Clavien mean [CI]: 5.3 ± 10.6; [4.1–6.5], *p* = 0.102).

Sample size calculations (for a theoretical trial with the assumptions as indicated in Fig. 3) for each procedure based on the traditional Clavien system or CCI revealed that using the CCI would result in considerably smaller sample sizes for each procedure (Table 3).

**Table 3:** Sample size calculation and assumption for each procedure

Procedure		Assumption	Sample size (per group)
RP cohort			
No. of patients with CDC complication	87/291 = 29.3%	30% reduction in complication	378
Mean CCI ± SD	5.3 ± 10.6	Δ 5; SD 10.6	77
PN cohort			
No. of patients with CDC complication	63/213 = 29.6%	30% reduction in complication	372
Mean CCI ± SD	7.0 ± 11.8	Δ 5; SD 11.8	95
RC cohort			
No. of patients with CDC complication	133/172 = 77.3%	30% reduction in complication	65
Mean CCI ± SD	20.4 ± 16.7	Δ 10; SD 16.7	49

*CCI* comprehensive complication index, *CDC* Clavien–Dindo-Classification, *No.* Number, *PN* partial nephrectomy, *SD* Standard deviation, *RP* radical prostatectomy, *R*C radical cystectomy

## Discussion

This study introduces the CCI for uro-oncological procedures using a large patient cohort at a tertiary university referral center. The cumulative CCI can be used to represent the total burden of postoperative complications and serves as a better predictor of postoperative LOS than the Clavien system for RC. Application of the CCI as a measure of complications in future trials would reduce the number of patients needed to identify relevant treatment effects and should be used with non-parametric testing for statistical analyses.

The CCI was initially introduced in general surgery and has become increasingly adopted as a clinical endpoint in surgical trials [16–18]. It has additionally been validated and implemented for various procedures such as gastric cancer surgery [19], hyperthermic intraperitoneal chemotherapy [7], and natural orifice transluminal endoscopic surgery [20]. The current standard for urology remains the Clavien system [4]. Vetterlein et al. provided a thorough analysis of complications following RC using the CCI as an adjunct measure [10]. They found comparable results (mean non-cumulative CCI: 21; mean cumulative CCI: 29) to our study (mean non-cumulative CCI: 21 mean cumulative CCI: 27). Furrer et al. proposed a modified Berne CCI specifically for RC since complicated postoperative courses can also result in a CCI of 99.4 for non-death cases [9]. The Berne CCI was able to predict death between postoperative day 90 to 1 year. Out of our cohorts the CCI yielded the most benefit for RC, which is unsurprising considering its complexity with advanced resection and reconstruction. Almost 50% of patients undergoing RC experienced more than one complication, and since the CCI accounts for all complications rather than just those of the highest-Clavien grade, one third of RC patients were re-classified to a higher Clavien grade. This is consistent with the fact that the CCI provided a more accurate prediction of LOS compared to the traditional Clavien system. In contrast, RP and PN generally have lower complication rates, especially in the era of robotic-assisted surgery. Hence, the CCI did not differ from the Clavien system for the majority of patients undergoing these procedures. However, differences were demonstrated for 16% of PN and 9.4% of RP patients, which is still remarkable. Furthermore, the CCI might be used to further analyze the impact of the surgeon’s experience [21] and the role of hospital volume [22, 23] on perioperative outcomes. These factors are increasingly considered and might help to predict the potential impact of centralization after major cancer surgery in urology [24]

Aside from the more comprehensive overall assessment, an additional benefit of the CCI is the reduction of required sample sizes in clinical trials. In general, the CCI is a more sensitive measure to identify slight differences compared to the Clavien system. In order to calculate sample size based on the Clavien system, total complications are often dichotomized to either occurrence (yes/no) or severity (minor/major). This results in the loss of pertinent information that can otherwise be accounted for with the CCI through its weighted complication estimates. Slankamenac et al. supported the aforementioned benefit by using the CCI to demonstrate a sample size reduction in a pancreatic and esophageal surgery clinical trial [14]. Since the CCI is simple to calculate and based on the well-known Clavien system, there should be minimal difficulty in implementation. Coupling this with a decrease in sample size requirement and clinical trials become more feasible, which is of paramount importance considering nearly 70% of phase III oncology trials fail to reach their planned sample sizes and 30% are terminated early due to low accural [25, 26]. Furthermore, this would allow for minor differences between slightly different surgical techniques to be detected for both retrospective and prospective cohorts. In summary, the CCI provides a more complete analysis of postoperative complications and thereby depicts a more accurate mean severity of postoperative complications for both individual patients and cohorts.

In terms of data distribution, normality of the CCI cannot be assumed for all three procedures based on samples from the current cohort (RC, RP, and PN). This is important to consider since plans for statistical analysis in clinical trials should be defined a priori. Furthermore, preliminary testing may result in uncontrolled error rates which is discussed in detail elswhere [12]. On the other hand, it has been shown that the parametric *t* test remains viable with large sample sizes even if the assumption of normality is violated [27, 28]. When in doubt, the use of non-parametric tests such as the Mann–Whitney–*U* test for group comparison appears adequate [12].

## Limitations

One limitation of the present study is that the CCI is based on the Clavien system which has several limitations. For example, complications of different morbidities may be classified similarly e.g., stenting of hydronephrosis and radical nephrectomy may both be performed under general anesthesia thereby classifying each as IIIB. Furthermore, grading of the same complications may differ based on patient preference of hospital-workflow e.g., the same intervention done under general vs. local anesthesia. These issues were already discussed in detail by Rassweiler et al. [29, 30]. However, these limitations are limitation of the Clavien and should not be considered as limitations of the CCI. Moreover, it is possible that not all minor complications are recorded during data acquisition, which would underestimate the true number of complications. In addition, outpatient information were not included, which might also leads to an underestimation of the real burden of complications. However, as shown for the RC cohort, the merits of the CCI are best seen when patients have more than one complication, so future studies should attempt to capture all surgical complications in order to provide better data quality. In doing so, a more accurate comprehensive analysis will better represent the overall complication burden. However, neither the CCI nor the Clavien system consider intraoperative complications. However, the “Intraoperative Adverse Incident Classification” was recently introduced to assess intraoperative complications [31]. Moreover, future studies might also involve patient opinions and views as well as quality of life in relation to perioperative complications.

Furthermore, as compared to other countries, the use of robotic-assisted PN is underrepresented in Germany, mainly due to reimbursement issues. This becomes obvious looking the study by Flegar et al. who compared trends in renal tumor surgery between the United States and Germany and might also influence the LOS [32].

Lastly, additional procedures such as retroperitoneal lymphadenectomy or radical nephrectomy were not included and may be subject of future studies.

## Conclusions

The CCI serves as a valuable metric for more accurately assessing postoperative complications in the three most common uro-oncological procedures. Particularly for RC, the CCI provided a significant comprehensive overview of the true complication burden. It can reduce required sample sizes and thereby facilitate future clinical research. Due to a skewed data distribution, statistical analysis via non-parametric testing is recommended.

## Electronic supplementary material

Below is the link to the electronic supplementary material.

Supplementary Figure Q-Q-plots to test for normality (JPG 95 kb)
