# Supplementary figures and images for: The comprehensive complication index (CCI): proposal of a new reporting standard for complications in major urological surgery
Source: World J Urol. 2020 Aug 19;39(5):1631–9. doi: 10.1007/s00345-020-03356-z (PMC8166677; doi:10.1007/s00345-020-03356-z)

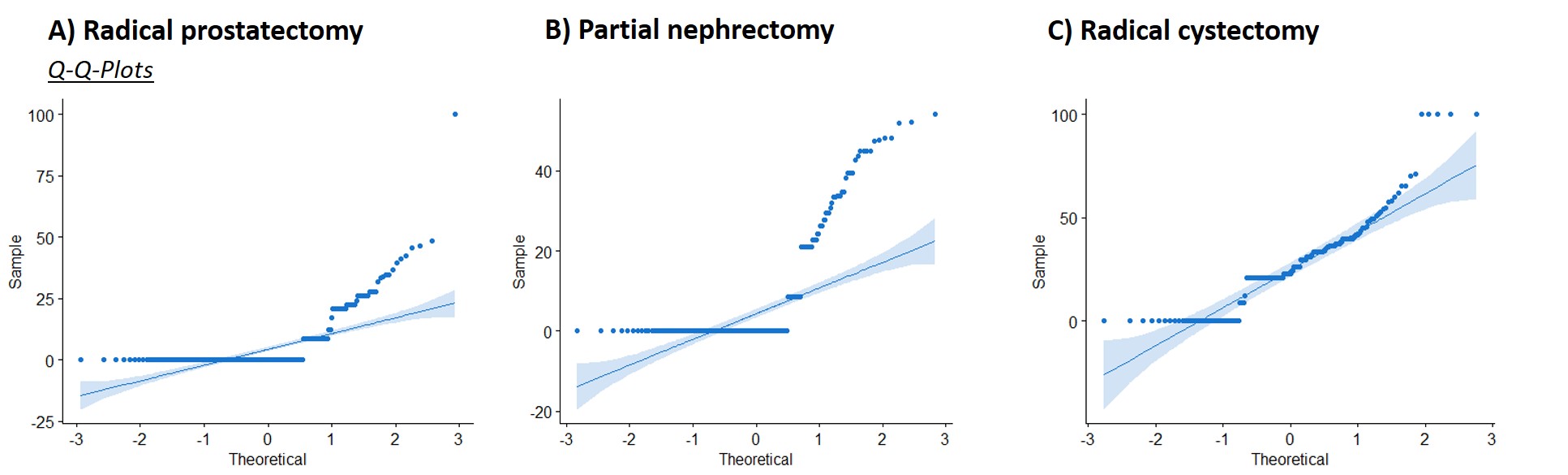

Supplement: Supplementary file 1 — Supplementary Figure Q-Q-plots to test for normality (JPG 95 kb) [file 345_2020_3356_MOESM1_ESM.jpg]
